# Correlation between craniofacial growth and upper and lower body heights in subjects with Class I occlusion

**DOI:** 10.1590/2177-6709.23.2.037-045.oar

**Published:** 2018

**Authors:** Thikriat S. Al-Jewair, Charles Brian Preston, Carlos Flores-Mir, Paul Ziarnowski

**Affiliations:** 1 State University of New York at Buffalo, Department of Orthodontics (New York, USA).; 2 University of Alberta, Faculty of Medicine and Dentistry, School of Dentistry, Division of Orthodontics (Edmonton, Canada).

**Keywords:** Skeletal maturity, Height, Mandible, Craniofacial growth

## Abstract

**Objective:**

To correlate skeletal age, standing height, upper and lower body lengths, and selected craniofacial growth features in a sample of growing individuals, and to model craniofacial growth using multivariate regression.

**Methods:**

This was a retrospective cross-sectional study with 447 African black boys and girls, between the ages 8 and 16 years, who attended the dental clinic at one hospital. The skeletal maturational age was determined from hand-wrist radiographs using the Greulich and Pyle atlas. Craniofacial measurements representing maxillary length (Ar-ANS), mandibular length (Ar-Gn), and lower facial height (ANS-Me) were calculated from lateral cephalograms in habitual occlusion. Body lengths were clinically measured in centimeters.

**Results:**

Moderate correlations (r=0.42 to 0.68) were observed between skeletal age and the three selected craniofacial measurements. Statistically significant correlations were also found between the craniofacial measurements and both upper and lower body lengths. The mandibular length had a stronger correlation with the upper body length than with the lower body length. Multiple regression analyses to determine maxillary and mandibular lengths suggested that sex, upper and lower body lengths might be used to determine maxillary length; while skeletal age, upper and lower body lengths might help determine mandibular length.

**Conclusions:**

Based on the relatively strong correlation between upper body length and mandibular length, further research in this area may warrant its use as a predictor for mandibular growth modification timing.

## RESUMO

**Objetivo:**

correlacionar a idade esquelética, a estatura, as alturas corporais superior e inferior, e algumas características específicas do crescimento craniofacial, em uma amostra de indivíduos em crescimento, e delinear o crescimento craniofacial usando regressão multivariada.

**Métodos:**

esse estudo transversal retrospectivo foi feito com 447 meninos e meninas negros africanos, com idades entre 8 e 16 anos, atendidos na clínica odontológica de um hospital. A maturação esquelética, em anos, foi determinada a partir de radiografias de mão e punho, usando o atlas de Greulich e Pyle. As medidas craniofaciais representando o comprimento maxilar (Ar-ENA), o comprimento mandibular (Ar-Gn) e a altura facial anterior inferior (ENA-Me) foram calculadas a partir de cefalogramas laterais em oclusão habitual. Os comprimentos corporais foram medidos clinicamente, em centímetros.

**Resultados:**

correlações moderadas (r = 0,42 a 0,68) foram observadas entre a idade esquelética e as três medidas craniofaciais selecionadas. Também foram encontradas correlações estatisticamente significativas entre as medidas craniofaciais e as alturas corporais superior e inferior. O comprimento mandibular teve uma correlação mais forte com a altura corporal superior do que com a inferior. As análises de regressão múltipla para determinar os comprimentos maxilar e mandibular sugeriram que o sexo e as alturas corporais superior e inferior podem ser usados para determinar o comprimento maxilar, enquanto a idade esquelética e as alturas corporais superior e inferior podem ajudar a determinar o comprimento mandibular.

**Conclusões:**

com base na correlação relativamente forte entre a altura corporal superior e comprimento mandibular, pesquisas adicionais nessa área poderiam justificar seu uso como indicador para o período de modificação do crescimento mandibular.

## INTRODUCTION

One of the treatment modalities in Orthodontics involves the use of orthopedic appliances in an attempt to modify dentofacial skeletal growth. These appliances appear to be more effective when used around adolescent skeletal growth spurt.^1^

Several skeletal growth indicators have been used to identify the periods of acceleration, spurt, and deceleration during adolescent skeletal maturation. These include standing height, changes in upper and lower body proportions, secondary sexual maturational characteristics, dental development, insulin-like growth factor-1 (IGF-1) and IGF binding protein-3, and radiographic assessment of skeletal maturation using cervical vertebrae or hand-wrist.^2^ ^-^ ^7^ The hand-wrist radiographs are still considered the reference standard in predicting overall skeletal maturation.^8^

It has been established that upper and lower body components showcase different patterns of growth.^9^ The peak in growth of the lower body (i.e., long bones) occurs on average one year before the peak in growth of the upper body (i.e., vertebral column);^8^ while increments in the upper body are larger than those of the lower body.^8^ ^,^ ^10^

Changes in standing height could be used with some success to imply the occurrence of craniofacial growth spurt^11^. Nonetheless, only one study has correlated changes in upper and lower body dimensions with craniofacial growth. Cozza et al^12^ investigated this correlation in the pre-pubertal period on a sample of Caucasian patients using clinical anthropometric measurements. They concluded that none of the body measurements was accurate indicator of craniofacial growth during the pre-pubertal phase. No follow up study is available to assess the potential strength of those correlations during adolescence. Therefore, the aims of the present retrospective study were to: 1) evaluate the correlation between skeletal maturation (hand-wrist radiographs), upper body length (sitting height), lower body length (leg length), and selected cephalometric measurements among African blacks aged 8-16 years; and 2) identify if upper and lower body lengths can independently determine craniofacial growth during adolescence.

## MATERIAL AND METHODS

The study was approved by the University at Buffalo Health Sciences Institutional Review Board. This retrospective cross-sectional study examined the correlation between different anthropometric measurements from the records of 447 (228 females and 219 males) African black urban children with chronological ages between 8-16 years^13^ (females: 12.1±2.3 and males: 12.5±2.1 years old). The subjects were randomly selected patients attending the dental clinic at one hospital. Assent was obtained from parents of all children before data collection. Records collected included demographics (chronological age and sex), left hand-wrist radiographs, lateral cephalograms, standing height in centimeters, sitting height, and body mass in kilograms. The inclusion criteria were healthy and well-nourished boys and girls, with Class I skeletal and dental occlusion; no previous orthodontic treatment received or required. The exclusion criteria were the presence of syndromes, systemic diseases such as metabolic bone diseases, developmental disturbances, long-term medications, or premature loss of permanent teeth. 

The left hand-wrist radiographs were obtained using the same machine and with a standardized target to film distance of 75 centimeters. The second edition of the Greulich and Pyle atlas^14^ was used to determine the skeletal maturational age. This method (GP) uses series of standard plates to be compared with the subjects’ hand-wrist radiographs so that their maturational age using maturity indicators of individual ossification centers is obtained. The values assigned to each ossification center were used to determine an average skeletal age of each subject. One calibrated operator who was blinded to the chronological ages conducted all measurements. All the records were de-identified at the time of data collection. 

Systematic error was assessed using 40 randomly selected radiographs with equal sex distribution obtained from the GP atlas by an independent observer. The mean error was 4.1 ± 3 months (standard error of the mean = 0.84 months, t-value = 0.68, 0.05 > P > 0.01). Intra-operator error was calculated using 39 radiographs (17 males and 22 females) obtained from the sample collected for this study. The mean error was -0.36 ± 5.90 months (standard error of the mean = 0.97 months, t-value = 0.39, P < 0.05). 

Lateral cephalograms in habitual occlusion were obtained using the same cephalostat with an exposure set between 86 to 93 Kv and a target to film distance of five feet. The magnification percentage of radiographs was 5.8%. One author manually traced and analyzed the cephalograms using a number of landmarks. Measurements used to the nearest 0.5 mm were the lower anterior facial height (LAFH), measured between the anterior nasal spine (ANS) and menton (Me); the mandibular unit length, measured from Articulare (Ar) to Gnathion (Gn); the maxillary unit length, measured from Ar to ANS.^15^ ^,^ ^16^ The center of the section where the ANS is 3mm in thickness was used. Intra-operator error was less than 1% and not statistically or clinically significant. 

Standing height (stature) was measured by using the horizontal arm of the anthropometer on a clinical scale. The subjects were instructed to stand with their heels together, soles in contact with the platform, stretching upward to the fullest extent, aided by the measurer, who exerted gentle pressure on the mastoid processes. The subject’s back was kept straight as possible, with the Frankfurt plane parallel to the floor. The recording arm of the anthropometer was then lowered on the subject’s head. Measurements were taken to the nearest 1 mm, with the horizontal arm lightly in contact with the child’s head. Contact of the measuring arm was made as close to the midsagittal plane of the skull as possible, and tangent to the highest point of the head in the median plane. 

Upper body length (sitting height) in centimeters included trunk length plus head length (vertex to subischial plane). A bench was placed over the platform of the clinical scale, of such a height that it allowed the subjects to sit with their unsupported feet hanging over the edge. Measurements were taken with the child’s back stretched up straight and with the back of the knees directly over the edge of the seat. Extension of the spine was encouraged in the same way as for the standing height, while the head was postured in the Frankfurt plane. The bench was 35 cm in height and was subtracted from the total sitting height. 

Lower body length (leg length) in centimeters (femur plus tibia) was obtained by subtracting standing from sitting height, to compare growth of long bones to upper body, including vertebral column. 

### Statistical analysis

Descriptive statistics for all variables were calculated. Independent samples t-test was used to identify differences between females and males in the body lengths and craniofacial measurements at each skeletal age (rounded to the nearest year). Normal data distribution was observed. Pearson correlation coefficients^17^ were calculated for the chronological and skeletal ages, standing height, upper and lower body lengths, and craniofacial measurements. Correlations were two-tailed and interpreted at the 1% and the 5% significance levels, with the Bonferroni adjustment applied for multiple testing. A stepwise multiple linear regression with backward elimination was used to determine predictors of craniofacial growth patterns, while controlling for chronological and skeletal ages, sex, and upper and lower body lengths. Data were analyzed using PASW for Windows version 20.

## RESULTS

**Figure 1:**
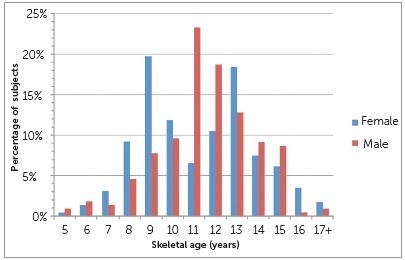
Percentages of subjects at various skeletal ages.

The skeletal ages of the sample using the GP method ranged from 5 to 17+ years old (Table 1). The mean skeletal ages were 11.6 ± 2.6 years for females and 12.0 ± 2.3 years for males. A breakdown of the skeletal ages by sex is depicted in Figure 1. 

**Table 1:** Cross-tabulation of chronological and skeletal ages

		Skeletal age by year														Total
		up to 4.9	5	6	7	8	9	10	11	12	13	14	15	16	17+	
Chronological age by year	16												2			2
	15									2	10	16	22	6	3	59
	14							2	2	19	28	17	5	3	1	77
	13						1	1	7	18	20	3	2		1	53
	12						3	2	26	20	11					62
	11				1		6	17	19	4	1	1	1		1	51
	10			1		8	25	18	7	1						60
	9				2	17	25	5	5							54
	8	3		6	7	6	2	3		1			1			29
Total	3		7	10	31	62	48	66	65	70	37	33	9	6	447	

Table 2 presents the differences in standing height between males and females at various skeletal ages. A large difference between females and males was seen at skeletal age 11 (6.06 cm) and was statistically significant (P=0.003) both at the nominal level of 0.05 and at the Bonferroni adjusted level of 0.01, based on tests for eight skeletal age groups with sufficient numbers of observations (30+) for reliable statistical testing. A relatively large difference was also seen at age 15 (3.39 cm), but the finding was not statistically significant after adjustment (P=0.047). There was a general gradual increase in leg length for both sexes as skeletal age group increased. A statistically significant difference of 2.62 cm was noted between females (64.38 cm) and males (61.76 cm) at age 9 (P=0.023). Similarly, the 3.56 cm difference at age 11 and 3.28 cm difference at age 15 (greater for males) were also statistically significant (P=0.026 and P=0.038, respectively). When the upper body length was measured, the largest difference between females and males was 2.5 cm at age 11. However this did not achieve statistical significance (P= 0.090).

**Table 2:** Mean standing height, sitting height, and leg length per skeletal age increment.

Age increment (years)	Standing height (cm)		Upper body length (cm)		Lower body length (cm)	
	Females	Males	Females	Males	Females	Males
	Mean ± SD	Mean ±SD	Mean ±SD	Mean ± SD	Mean ± SD	Mean ± SD
5	119.5^†^	116.3 ± 6.08	60.4^†^	62.9 ± 2.40	59.1^†^	53.4 ± 3.68
6	118.9 ± 6.61	121.5 ± 4.74	62.0 ± 3.66	62.5 ± 3.13	56.8 ± 3.23	59.0 ± 5.06
7	124.0 ± 4.49	123.9 ± 5.16	65.0 ± 3.09	65.5 ± 4.77	59.0 ± 2.15	58.4 ± 1.86
8	129.6 ± 4.34	129.1 ± 4.65	66.4 ± 2.13	67.1 ± 2.96	63.2 ± 3.81	62.0 ± 4.88
9	132.3 ± 6.15	130.6 ± 3.60	67.9 ± 3.19	68.9 ± 3.83	64.4 ± 4.08	61.8 ± 3.48*
10	135.0 ± 5.39	134.0 ± 4.47	69.6 ± 2.16	69.4 ± 2.77	65.3 ± 3.99	64.6 ± 3.41
11	144.6 ± 5.61	138.5 ± 6.93*	73.8 ± 4.23	71.3 ± 5.12	70.8 ± 2.88	67.2 ± 5.80*
12	144.8 ± 6.96	144.5 ± 7.26	74.0 ± 3.58	72.9 ± 3.09	70.8 ± 6.13	71.6 ± 6.79
13	150.9 ± 7.00	150.2 ± 5.73	76.0 ± 3.27	75.0 ± 2.79	74.8 ± 5.77	75.2 ± 4.59
14	154.1 ± 7.91	154.3 ± 10.20	77.5 ± 3.98	77.6 ± 3.86	76.6 ± 5.33	76.7 ± 8.81
15	155.5 ± 3.69	158.9 ± 5.23*	79.3 ± 1.96	79.4 ± 2.86	76.2 ± 3.37	79.5 ± 4.84*
16	158.0 ± 9.60	158.2^†^	80.2 ± 5.46	75.5^†^	77.8 ± 4.63	82.7^†^
17+	152.7 ± 6.85	163.5 ± 8.56	76.9 ± 2.90	81.3 ± 5.23	75.8 ± 6.54	82.2 ± 3.32

* P<0.05; statistically significant difference between females and males using two tailed tests.

† One observation, standard deviation (SD) undefined.

**Figure 2:**
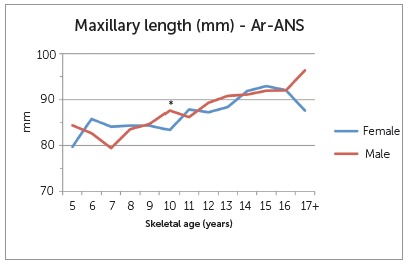
Differences in maxillary lengths (Ar-ANS) at various skeletal ages.

**Figure 3:**
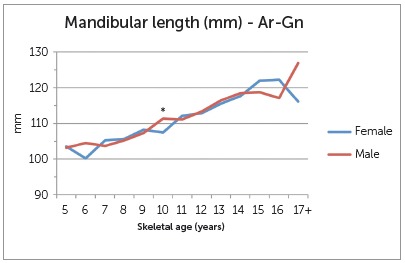
Differences in mandibular lengths (Ar-Gn) at various skeletal ages.

**Figure 4:**
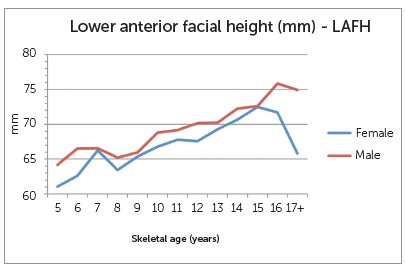
Differences in lower anterior facial heights at various skeletal ages.

Craniofacial measurements at the different skeletal ages are depicted in Figures 2, 3 and 4. There was a general gradual increase in maxillary length for both sexes as age group increased. There were large differences between females and male groups at the early ages (5, 6 and 7 years old) and older ages (17+), as well as differences within the groups that represented as a decrease in maxillary length with increasing age. Nonetheless, these groups had very small representation and, as such, statistical testing was unreliable. The difference at age 10 however, showed statistical significance (4.27 mm, P=0.002). For mandibular length, a statistically significant difference was also seen at age 10 (3.86 mm, P=0.002). No other age groups demonstrated a significant difference between females and males. Results of the LAFH revealed a statistically significant difference of 2.60 mm at skeletal age 12 (P=0.041) at the nominal level of 5%, but not for the Bonferroni adjusted cut-off of 1%. The difference of 9.12 mm seen for the age group 17+ was also statistically significant (P=0.031), but the number of observations was too small for reliable statistical testing. 

Results of the linear correlations between the skeletal ages for females and males, the standing height, the upper and lower body lengths, and the craniofacial measurements are presented in Table 3. The correlation between the skeletal ages and the chronological ages was stronger for females (r=0.91) than for males (r=0.78). Moderate correlations (r=0.42 to 0.68) were noted between the skeletal age and the craniofacial measurements. Similar correlations were also found between both, upper and lower body lengths, and the craniofacial measurements. Correlations of the mandibular length and lower facial height were stronger with the upper body length than with the lower body length. 

**Table 3:** Pearson correlation coefficients (r) between skeletal maturational indicators and craniofacial measurements.

	Sex	Chronological age (years)	Skeletal age (years)	Standing height (cm)	Upper body length (cm)	Lower body length (cm)	Ar-ANS (mm)	Ar-Gn (mm)	LAFH (mm)
Chronological age (years)	M	1	0.78**	0.71**	0.59**	0.64**	0.39**	0.52**	0.43**
	F	1	0.91**	0.82**	0.77**	0.75**	0.40**	0.64**	0.44**
Skeletal age (years)	M	0.78**	1	0.83**	0.71**	0.73**	0.48**	0.62**	0.42**
	F	0.91**	1	0.85**	0.82**	0.76**	0.44**	0.68**	0.44**
Standing height (cm)	M	0.71**	0.83**	1	0.79**	0.92**	0.51**	0.64**	0.40**
	F	0.82**	0.85**	1	0.91**	0.95**	0.49**	0.74**	0.51**
Upper body length (cm)	M	0.59**	0.71**	0.79**	1	0.49**	0.43**	0.59**	0.41**
	F	0.77**	0.82**	0.91**	1	0.72**	0.49**	0.72**	0.47**
Lower body length (cm)	M	0.64**	0.73**	0.92**	0.49**	1	0.46**	0.54**	0.31**
	F	0.75**	0.76**	0.95**	0.72**	1	0.43**	0.67**	0.47**
Ar-ANS (mm)	M	0.39**	0.48**	0.51**	0.43**	0.46**	1	0.65**	0.22**
	F	0.40**	0.44**	0.49**	0.49**	0.43**	1	0.61**	0.32**
Ar-Gn (mm)	M	0.52**	0.62**	0.64**	0.58**	0.54**	0.65**	1	0.56**
	F	0.64**	0.66**	0.74**	0.72**	0.67**	0.61**	1	0.64**
LFH (mm)	M	0.43**	0.42**	0.40**	0.41**	0.31**	0.22**	0.56**	1
		0.44**	0.44**	0.51**	0.47**	0.47**	0.32**	0.64**	1

** Correlation is significant at the 0.01 level (2-tailed).

* Correlation is significant at the 0.05 level (2-tailed).

Tables 4 and 5 present the stepwise multiple regression analyses with backward reduction. The models suggested that sex, upper and lower body lengths might be used to predict maxillary length; while skeletal age, upper and lower body lengths might help predict mandibular length. 

**Table 4:** Stepwise multivariate linear regression with backward elimination for the association between maxillary length (Ar-ANS) and different growth indicators.

Variable	β Coefficient	SE.	Standardized β	t	P	95% CI		R^2^	Adjusted R^2^
						Lower limit	Upper limit		
Sex	1.07	0.48	0.09	2.22	0.027	0.12	2.01	0.27	0.26
Upper body length (cm)	0.28	0.07	0.26	4.00	<0.001	0.14	0.41		
Lower body length (cm)	0.16	0.05	0.21	3.42	0.001	0.068	0.25		

**Table 5:** Stepwise multivariate linear regression with backward elimination for the association between mandibular length (Ar-Gn) and different growth indicators

Variable	β Coefficient	SE	Standardized β	t	P	95% CI		R^2^	Adjusted R^2^
						Lower limit	Upper limit		
Skeletal age (years)	0.56	0.23	0.19	2.45	0.015	0.11	1.01	0.51	0.50
Upper body length (cm)	0.46	0.07	0.36	6.79	<0.001	0.33	0.60		
Lower body length (cm)	0.22	0.046	0.23	4.68	<0.001	0.12	0.31		

## DISCUSSION

This study used the GP method to assess the skeletal ages of the subjects. The expected standard deviation of calculation errors in skeletal age assessments using the GP atlas is four to six months and it is best employed in cross-sectional studies.^18^ Its strength lies in the relative ease with which radiographs may be placed relative to a set of standards.^19^

This study revealed statistically significant differences in standing height between females and males at skeletal ages 11 and 15. Females showed higher standing height than males at earlier age (skeletal age 11; 6.06cm). At later age (skeletal age 15; 3.4cm), males surpassed females and became taller. These differences are in accordance with previous studies that showed that females experience an earlier pubertal growth spurt, then boys surpass girls at the older age.^20^ ^,^ ^21^

By eliminating the contribution of the lower body to standing height, the maximum upper body length was reached at more or less the time when the maximum standing height was attained, with no significant sexual dimorphism. A previous study used upper body length to predict standing height in patients with leg deformities.^9^ The lower body length, on the contrary, showed variability as compared to standing height and upper body length. Females showed earlier lower body length spurts than males, at ages 9 and 11. All these findings may suggest that changes in upper body length may be more valid for predicting maturational growth spurt than changes in lower body length. 

Overall, maxillary and mandibular lengths increased gradually with increasing skeletal age for both sexes. At skeletal age 10, however, males showed acceleration in growth of maxilla and mandible, compared to females. The LAFH in the sample of this study was greater than what has been reported for other racial groups. This relatively greater lower anterior facial dimension in the African black children is in agreement with Jacobson,^22^ who noted that South African blacks have increased mandibular plane angles (SN-MP). The LAFH also showed some sexual dimorphism. Males had significantly greater LAFH than females at skeletal age 12. This is following the same trend observed for the maxilla and mandible, and in accordance with earlier studies that concluded that males have more growth increment and duration, compared to females.^6^ ^,^ ^23^

Similar to previous studies,^24^ ^,^ ^25^ mandibular length in this study showed a statistically significant correlation with skeletal age in both females and males. Yet the clinical significance of this correlation might be questioned. Individual variability may at least partially explain this. The maxilla on the other hand showed a variable level of growth with increasing skeletal age. This can be explained by the fact that it undergoes growth at the sutures in addition to the remodeling of bones, which might put it under the control of other factors, in comparison to the mandible.^24^ ^,^ ^26^

When correlated with upper and lower body lengths, mandibular length showed a stronger correlation with the upper body length, compared to the lower body length. The upper body length includes both the head and the vertebral column lengths. Although cranial growth slows down after age 5, the head height and width have shown slight acceleration during growth spurt.^27^ Thus it is possible that the growth of the vertebral column is mainly responsible for the substantial acceleration during adolescence. The cervical vertebrae make the upper portion of the vertebral column. The cervical vertebral maturation (CVM) and its relation with mandibular growth has long been suggested in many earlier studies.^25^ However, the CVM method has undergone criticism lately, due to its poor reliability and reproducibility. Ball et al^7^ reported variability in the timing of each CVM stage, with the average time spent in stage 4 being 3.79 years. They concluded that the CVM on itself couldn’t consistently predict pre-pubertal or peak of mandibular growth. Future studies are needed to further understand the relationship between mandibular growth and sitting height. 

On the multivariate level, this study found that the upper and lower body lengths were independent predictors of both the maxillary and mandibular lengths. This may have clinical implications in terms of choice of growth indicators. Taking clinical anthropometric measurements more consistently, in addition to radiographs, to predict the timing of orthopedic treatment may be something to be considered. For the maxillary prediction equation, sex is the most significant predictor. Ursi et al^28^ reported a significant difference in midfacial length (Co-PtA) between males and females with normal craniofacial pattern at age 14 and older, with males exceeding females in values. Baccetti et al^29^ also showed that females with Class III malocclusion exhibited shorter midfacial length (Co-Pt.A) than males at the pubertal and postpubertal age of 13 years and older. 

For the mandibular prediction equation in the present study, the skeletal age, the upper and the lower body lengths were relatively similar strong contributors, while sex was not a contributor. Previous studies^30^ indicated that sexual dimorphism was more evident for the corpus size and velocity than the ramus height. Thus, the sexual dimorphism in the total mandibular length is less clear. 

The results of this study cannot be generalized to other racial groups. Normal growth patterns of African black children are said to differ significantly from those of Caucasian children.^31^ ^,^ ^32^ These ethnic differences in growth appear to transcend family, social and economic backgrounds, since African black children from low income families grow faster and mature earlier than middle class white children.^33^

The cross-sectional data in this study did not allow the estimation of mean velocity peaks in body lengths or craniofacial dimensions as it would be possible with longitudinal studies^34^ nor was it set to determine cause and effect relationship. Previous studies have shown that the onset of pubertal growth in the upper body length starts on average at 75 cm in females and 78 cm in males,^10^ and an increase of up to 84 cm indicates menarche in 80% of the females.^10^ ^,^ ^35^ Also at the onset of puberty, boys and girls have 14% (22.5 cm ± 1; made up by 13 to 9.5 cm upper to lower body proportion) and 12% (20.5 cm ± 1; made up by 12 to 8.5 cm upper to lower body ratio) of their remaining standing height to grow. Therefore, it is recommended that future studies evaluate the growth changes and correlations prospectively. It is also worthwhile investigating longitudinally the association between the upper and lower body lengths with the mandibular ramus and corpus lengths, to increase our understanding of facial patterns of growth. 

## CONCLUSIONS

There were moderate correlations (r=0.42 to 0.68) between the three evaluated craniofacial measurements (Lower anterior facial height [ANS-Me]; mandibular unit length [Ar-Gn]; and maxillary unit length [Ar-ANS]) and skeletal age. The same measurements were also correlated (r=0.43 to 0.72) with the upper and lower body lengths. The mandibular length had a stronger correlation (r=0.58 in females and 0.72 in males) with the upper body length than with the lower body length. 

Multiple regression analyses suggested that sex, upper and lower body lengths may be useful partial predictors of maxillary length (adjusted r =0.26); while skeletal age, upper and lower body lengths might partially predict mandibular length (adjusted r =0.50). 
